# Age-dependent effects in the association between monetary delay discounting and risky sexual behavior

**DOI:** 10.1186/s40064-016-2570-1

**Published:** 2016-06-23

**Authors:** Jeb Jones, Patrick S. Sullivan

**Affiliations:** Emory University, 1518 Clifton Road, Atlanta, GA 30322 USA

**Keywords:** Delay discounting, Men who have sex with men, HIV, Condomless anal intercourse

## Abstract

**Background:**

Monetary delay discounting is a measure of impulsivity associated with substance use and abuse, problem gambling, and other health-related outcomes. More recently, delay discounting has been shown to be associated with risky sexual behavior. We analyzed survey data from men who have sex with men who completed a monetary discounting task and reported sexual behaviors in the previous 12 months.

**Findings:**

Monetary delay discounting was associated with condomless anal intercourse among young (18–24 years), but not older, men who have sex with men.

**Conclusions:**

Monetary delay discounting may identify young men at increased risk of engaging in HIV risk behaviors.

## Backgrounds

There is growing evidence that delay discounting is associated with sexual behavior and risk taking (Dariotis and Johnson 2015; Herrmann et al. 2014, 2015; Johnson and Bruner 2012; Johnson et al. 2015; Jarmolowicz et al. 2015; Jones and Sullivan 2015). Long known to be associated with substance abuse and problem gambling (Reynolds 2006), delay discounting is a measure of impulsivity that refers to individuals’ tendency to prefer small rewards available immediately or in the near future over larger rewards available at some later time. The subjective devaluation of rewards to be received at a delay has been shown to take a hyperbolic form (Mazur 1987), reflecting a tendency for preferences to shift from larger to smaller rewards as the delay to the small reward decreases. This is evident in substance abuse when, for example, an individual resumes cigarette smoking a day after resolving to quit smoking. The previously preferred larger later reward of better health has been traded for the smaller sooner reward of smoking the cigarette.

Similar processes might be at play in the context of risky sexual behavior. Men who plan to always use condoms might switch their preferences in the heat of the moment because a condom is not available or because their partner prefers condomless sex. In this case, the larger later reward of good sexual health and possible disease avoidance is traded for the smaller, sooner reward of a sexual encounter without a condom.

Delay discounting is frequently measured using a hypothetical monetary choice task in which participants select between smaller amounts of money available immediately versus larger amounts of money available at a specified delay. The pattern of responding for different amounts of money available at different delays permits the derivation of a discounting rate that fits the following hyperbolic equation:

1 $${\text{V}} = {\text{A/}}\left( {1 + {\text{kD}}} \right)$$

where *V* is the subjective present value of a given amount, *A*, of money available at a specified delay, *D* (Mazur 1987). The free parameter *k* describes the rate at which the value of a future reward is discounted. Higher values of *k* reflect higher rates of discounting and greater preference for immediate rewards.

A few studies have explored the relationship between delay discounting and sexual behavior. Whereas monetary delay discounting tasks focus on the preference between small amounts of money available after a short delay versus larger amounts of money available after a longer delay, the type of sexual activity to measure using delay discounting tasks is less clear. For example, some studies have focused on the delay to condom availability (e.g., Johnson and Bruner 2012). Other studies have examined discounting of the duration of sexual activity (i.e., shorter sexual episode now versus longer episode later; Lawyer et al. 2010) and have found the rate of discounting related to sexual discounting to be hyperbolic in form, similar to monetary discounting. The relationship between monetary discounting and sexual behavior remains unclear (Johnson and Bruner 2012; Jones and Sullivan 2015). Although Johnson and Bruner (2012) observed an association between monetary discounting and sexual behavior in the same direction as Jones and Sullivan (2015), the former was not statistically significant; however, this might be an artifact of the different sample sizes in the two studies. To the extent that it is associated with sexual risk taking, delay discounting has the potential to contribute to HIV prevention efforts. Measures of sexual, and potentially monetary, discounting have the potential to aid in the identification of individuals at high risk of engaging in risky sexual behavior, and therefore, HIV and STI incidence. Further, delay discounting is a potential target of behavioral interventions that could reduce an individual’s rate of discounting and propensity to engage in impulsive behavior (e.g., Bickel et al. 2011; Black and Rosen 2011).

We sought to examine whether the association of monetary discounting and sexual behavior varied by age. A number of studies investigating discounting and sexual behavior have been conducted with undergraduate populations, and it is unclear how well these results generalize to older adults. Specifically, the neural areas related to impulsivity continue to develop into the 20 s (Giedd 2004); thus, the relationship between measures of discounting and impulsive behavior might be different in younger versus older adults. That is, impulsive decision-making with regard to monetary outcomes might indicate a tendency to engage in impulsive or risky sexual behavior in young, but not older, adults. This is of particular interest because young men who have sex with men are currently the age group with the highest incidence of HIV infection in the United States (Prejean et al. 2011). There is an urgent need for intervention targets to reduce risky sexual behavior among young MSM and for methods to identify men most in need of HIV prevention intervention.

Using existing data from an online survey of MSM, we investigated the relationship between condomless anal intercourse (CAI), delay discounting, and age. Specifically, we were interested in whether the association between high delay discounting and CAI was different in young MSM (age 18–24) and older MSM (age 25 and above).

## Methods

### Participants

The survey methods and population have been described previously (Jones and Sullivan 2015). Briefly, an Internet-based survey was conducted of MSM recruited through Facebook advertisements in October and November 2011. Advertisements were targeted to men who reported being interested in men, were age 18 or older, and resided in the United States. Participants answered demographic questions, reported on their sexual behavior in the previous 12 months, and completed the monetary choice questionnaire (MCQ; Kirby et al. 1999). The MCQ is a series of 27 questions presenting a dichotomous choice between a given amount of money available immediately and a larger amount available at a delay. A respondent’s pattern of preferences is used to assign the delay discounting parameter, *k*. The survey took 5–10 min to complete and participants were not compensated for participation. The survey was reviewed and found to be exempt from review by the Emory IRB. Because this study was found to be exempt from IRB review and participants completed the survey anonymously, written informed consent was not obtained. In lieu of written informed consent, participants viewed a disclosure statement informing them about the survey and then decided whether to participate. The disclosure statement is available for review by the Editor-in-Chief of this journal.

### Analysis methods

The delay discounting parameter *k* was estimated for each participant based on his pattern of responding on the MCQ using the procedure described by Kirby et al. (1999). The *k* values were highly skewed and were log-transformed for analysis. There is no pre-specified criterion level that separates impulsive and non-impulsive delay discounting; as in the previous analysis (Jones and Sullivan 2015), we classified the top quintile of ln(*k*) values as high delay discounting (i.e., more impulsive).

We used a directed acyclic graph (DAG) approach to guide the modeling strategy and identify potential confounding variables. Based on the DAG, age (18–24 years old vs. 25 years or more), education (at least some college vs. high school or less), income (<$15,000/year vs. $15,000/year or more), and race (white vs. non-white) were determined to be possible confounders of the relationship between delay discounting and CAI. Drug use was hypothesized to most likely be an intervening variable between delay discounting and CAI and was therefore not entered into the model.

Log-binomial models were used to estimate the prevalence of high delay discounting in MSM reporting CAI compared to men reporting no CAI. Interactions between delay discounting and each of the hypothesized confounders were examined. A p value of <0.10 was considered statistically significant for the purposes of retention in the model. A backwards stepwise elimination procedure was used, first assessing the interaction terms followed by any single-order terms not contained in any statistically significant interaction terms.

We previously reported an association between high delay discounting and reporting 3 or more CAI partners in the previous 12 months based on these survey data (Jones and Sullivan 2015), but did not explore the possibility of interaction with age in that analysis. Therefore, we examined the outcome of 3 or more CAI partners in the current model to determine if age modified the association between delay discounting and CAI.

Because the delay discounting variable, *k*, is a continuous measure we also assessed the association between the log-transformed *k* and the prevalence of any CAI partners and the prevalence of 3 or more CAI partners.

## Results

Of 1562 survey participants, 1332 (85 %) provided data for each of the variables used in the current analysis. Participant characteristics are presented in Table 1. The age range of participants was 18–78 (median = 28, IQR: 21–44); 39 % of participants were under 25 years old. The participants were 77 % non-Hispanic white, 9 % Hispanic, and 2 % non-Hispanic black. The remaining 12 % reported some other race/ethnicity or identified as multiple races. 39 % of participants reported an annual income less than $15,000 and 79 % had at least some college education. High and low delay discounters differed with regard to income (p = 0.0216) and education (p = 0.0032) but not with regard to race or age. The upper quintile of delay discounting corresponded to an assigned *k* of 0.064 (ln*k* = −2.75) and the log-transformed *k* was approximately normally distributed.

**Table 1:** Demographic and behavioral characteristics of study population stratified by high and low discounting

Variable	High delay discounting N (%)	Low delay discounting N (%)
Age		
18–24 years	109 (40.2)	416 (39.2)
25 years or more	162 (59.8)	645 (60.8)
Race		
White	200 (73.8)	828 (78.0)
Non-white	71 (26.2)	233 (22.0)
Income*		
$0–$14,999/year	121 (44.7)	393 (37.0)
$15,000/year or more	150 (55.4)	668 (63.0)
Education*		
High school or less	76 (5.71)	210 (19.8)
At least some college	195 (72.0)	851 (80.2)

* Indicates p < 0.05

### Categorized delay discounting

The final model (Table 2) contained education, income, and the interaction of age by delay discounting. No other interaction term was statistically significant. Among men age 18–24, there was an 18 % higher prevalence of high delay discounters among those reporting CAI in the past 12 months (PR = 1.18, 95 % CI 1.05, 1.32), controlling for education and income. There was no statistically significant association between monetary discounting and CAI among men age 25 and older (PR = 0.95, 95 % CI 0.84, 1.08).

**Table 2:** Categorized delay discounting variable

Variable	<25 years old		≥25 years old	
	PR (95 % CI)	p	PR (95 % CI)	p
Any condomless anal intercourse				
Delay discounting^a^				
Low	Ref		Ref	
High	1.18 (1.05, 1.32)	<0.01	0.95 (0.84, 1.08)	0.46
Condomless anal intercourse with 3 or more partners				
Delay discounting^a^				
Low	Ref		Ref	
High	2.37 (1.62, 3.46)	<0.01	1.06 (0.76, 1.48)	0.74

Adjusted prevalence ratios (PR) and confidence intervals for condomless anal intercourse with any and multiple partners in the past 12 months

^a^Adjusted for education and income

The interaction between delay discounting and age was also observed when the outcome was 3 or more CAI partners versus 2 or fewer CAI partners. Among older MSM, the PR for high delay discounting was not statistically significant (PR = 1.06, 95 % CI 0.76, 1.48); the PR among young MSM (PR = 2.37, 95 % CI 1.62, 3.46) was stronger than that observed for one or more CAI partners, controlling for income and education.

### Continuous delay discounting

The results of modeling the continuous delay discounting variable are presented in Table 3. When modeling the continuous, log-transformed *k* value, there was a 3 % (PR = 1.03, 95 % CI 1.00, 1.07) increase in the prevalence of reporting at least one CAI partner for each one-unit increase in ln*k* among young MSM. No increase in the prevalence of reporting at least one CAI partner was observed among older MSM (PR = 0.99, 95 % CI 0.96, 1.02).

**Table 3:** Continuous delay discounting variable

Variable	<25 years old		≥25 years old	
	PR (95 % CI)	p	PR (95 % CI)	p
Any condomless anal intercourse				
Delay discounting^a^				
One-unit increase in ln*k*	1.03 (1.00, 1.07)	0.08	1.00 (0.96, 1.02)	0.43
Condomless anal intercourse with 3 or more partners				
Delay discounting^a^				
One-unit increase in ln*k*	1.27 (1.12, 1.45)	<0.01	1.00 (0.92, 1.08)	0.92

Adjusted prevalence ratios (PR) and confidence intervals for condomless anal intercourse with any and multiple partners in the past 12 months

^a^Adjusted for education and income

For the outcome of 3 or more CAI partners, there was a 27 % increase for each one-unit increase in ln*k* among young MSM (PR = 1.27, 95 % CI 1.12, 1.45). No increase in multiple CAI partners was observed among older MSM (PR = 1.00, 95 % CI 0.92, 1.08).

## Discussion

We observed an interaction between monetary delay discounting and CAI. Specifically, monetary delay discounting was associated with CAI among young MSM, but not among older MSM. Monetary delay discounting might identify young MSM at increased risk of HIV and provide a behavioral target for intervention development to reduce risky sexual behavior. The finding of an interaction between high delay discounting and age is also interesting because it might indicate developmental differences between younger and older MSM. That is, the MCQ might be measuring a broader, generalizable impulsivity among young MSM and a monetary-specific impulsivity among older MSM. Domain-specific sexual discounting tasks have been developed and it will be interesting to observe how these perform in the two different age groups of MSM.

We previously observed an association between high delay discounting and multiple CAI partners using these data, but we did not explore the possibility of an interaction between age and delay discounting in the earlier work. In a model using an outcome of multiple (≥3) CAI partners, the interaction remained significant, with a higher PR among younger MSM compared to the any-CAI-partners model. This suggests that the young MSM were responsible for the association we observed between risky sexual behavior and CAI in our previous analysis.

Although drug use has been consistently shown to be associated with delay discounting and risky sexual behavior, we did not include it in the current model because we hypothesized that it would likely serve as an intervening variable between discounting and CAI. We examined the effect of including drug use in the model and it did not change the estimate associated with delay discounting.

Our primary goal in this analysis was to examine the effect of age on a previously reported association. However, because the delay discounting variable, *k*, is continuous in nature, we also examined the association between delay discounting and sexual risk-taking using the log-transformed *k* value. The results of log-binomial models using ln*k* were consistent with the dichotomized delay discounting models. For the outcome of any CAI partners, there was a 3 % increase in the prevalence of reporting at least one CAI partner for each one-unit increase in ln*k*. Although this PR was not statistically significant, this corresponds to a 26 % increase in the prevalence of CAI comparing men with the steepest discounting functions to those with the shallowest discounting functions. As with the categorized delay discounting variable, the results were more pronounced for the outcome of 3 or more CAI partners. There was a statistically significant 27 % increase in reporting 3 or more partners for each one-unit increase in ln*k* among young men and no effect among older men. Future studies should explore more fully the form of the relationship between delay discounting and CAI and the effect of age on the association.

This analysis has limitations. This is a secondary analysis of data evaluating a post hoc hypothesis (Jones and Sullivan 2015). The data are derived from a cross-sectional study, and thus we are unable to speculate about the temporal relationship between delay discounting and CAI. Additionally, these results are not generalizable to other populations because they represent data collected from a convenience sample of MSM. Men who opt to participate in an uncompensated survey might differ with regard to monetary delay discounting compared to men who do not.

Due to the constraints imposed by an uncompensated, online survey, we were unable to ask about a number of variables that might influence sexual risk-taking. For example, participants reported whether they had engaged in anal intercourse without a condom, but they were not asked about other methods of HIV prevention. However, these data were collected prior to the approval of PrEP for HIV. We do not have information on partner type (e.g., main vs. casual) or perceived partner serostatus of CAI partners. However, the outcome of CAI is a risky behavior regardless of partner type and likely regardless of perceived partner serostatus (Golden et al. 2008).

Monetary delay discounting tasks are a promising tool for identifying individuals at high risk of engaging in risky sexual behavior. Such tasks might suffer from less social desirability bias because there is no clear ‘correct’ answer (Odum 2011), possibly resulting in higher sensitivity than other self-report measures of HIV risk. Further, to the extent that delay discounting is associated with risky sexual behavior, interventions designed to reduce discounting might lead to a reduction in sexual risk-taking. Based on the results of the current analysis, the utility of monetary discounting tasks to identify high-risk MSM is highest among young MSM.

The current analysis cannot identify a causal relationship between high delay discounting and CAI; however, it is plausible that high delay discounting results in impulsive decision-making such as engaging in risky sexual behavior. A longitudinal study is required to assess the causal role that delay discounting plays in risky sexual behavior among young MSM.

## Abbreviations

MSM: men who have sex with men
CAI: condomless anal intercourse
DAG: directed acyclic graph
